# ‘Unravelling the shared genetic architecture between suicidality and subcortical brain volume: a genome-wide association study’

**DOI:** 10.1017/neu.2025.12

**Published:** 2025-03-31

**Authors:** Joel Defo, Raj Ramesar

**Affiliations:** MRC Research Unit for Precision and Genomic Medicine, Division of Human Genetics, Department of Pathology, Institute of Infectious Disease and Molecular Medicine, Faculty of Health Sciences, University of Cape Town, South Africa

**Keywords:** Suicidality, brain, genomic SEM, genome-wide association studies meta-analysis, genes, pathway

## Abstract

Suicidality is a significant public health concern, with neuroimaging studies revealing abnormalities in the brains of suicidal individuals and post-mortem samples. However, the genetic architecture between suicidality and subcortical brain volumes remains poorly characterized. Using genome-wide association studies (GWAS), we investigated the genetic overlap between suicidality and subcortical brain volume. GWAS summary statistics for suicidal behaviours, including Suicide Attempts, Ever Self-Harmed, and Thoughts of Life Not Worth Living, from the UK Biobank, Suicide from the FinnGen Biobank, and data on seven subcortical brain volumes and Intracranial Volume from the ENIGMA2 study, were used to investigate the genetic correlation between phenotypes as well as potential genetic factors. A common genetic factor was identified, comprising two categories: Suicide Attempt, Ever Self-Harmed, and Thoughts of Life Not Worth Living from the UK Biobank, and Suicide from FinnGen, Intracranial Volume, and subcortical brain volumes. Cross-phenotype GWAS meta-analysis of each category at variant, gene and subnetwork levels unveils a list of significant variants (P-value <5 × 10^−8^), and potential hub genes (P-value <0.05) of consideration. Network, pathway, and Gene Ontology analyses of these joint categories highlighted enriched pathways and biological processes related to blood-brain barrier permeability suggesting that the presence and severity of suicidality are associated with an inflammatory signature detectable in both blood and brain tissues. This study underscores the role of brain and peripheral blood inflammation in suicide risk and holds promise for developing targeted interventions and personalized treatment strategies to reduce suicidality in at-risk populations.

## Significant outcomes

Suicidality, subcortical brain volume and Intracranial volume of individuals of European ancestry shared the same genetic common factor. Additionally, there is a positive genetic correlation between Suicide from FinnGen and Intracranial brain volume. Gene Ontology analyses, pathways and biological processes encompassing these phenotypes highlight shared mechanisms related to an inflammatory signature detectable in both blood and brain tissues.

## Limitations

This study solely focuses on individuals of European ancestry. Future studies should focus on including diverse ancestries for a better generalization. Additionally, this study had a limited sample size, which might have resulted in the inability to detect signals, improve genetic correlation, and detect shared biological mechanisms.

## Highlights

- Genomic Structural Equation Modeling analyses reveals a common latent factor divided into two distinct groups of phenotypes. The first group includes the suicidal traits from the UK Biobank (Ever Self Harm, Suicide Attempts, and Thought of Life Not Worth Living), while the second group includes Suicide from FinnGen, ICV, Accumbens, Caudate, Hippocampus, Pallidum, Thalamus, and Putamen from ENIGMA.
- Genome-wide association studies (GWAS) meta-analysis within the first group identifies 34 novel significant variants, mostly located within the *DCC, STIM2, MEAF6, and RSPO1* genes; 50 significant hub genes mostly expressed in brains tissues which include brain anterior cingulate cortex, cultured fibroblast cells, brain hippocampus, brain putamen basal ganglia, and brain substantia nigra.
- GWAS meta-analysis within the second group identifies 64 SNPs showing potential pleiotropic effects; 22 significant hub genes with top enriched tissues located in the heart and left ventricle, pancreas, putamen basal ganglia, substantia nigra, and hippocampus.
- The resulting network combining the hub genes from the two groups exhibit significant pathways connected to inflammatory signature detectable in both blood and brain tissues.

## Introduction

Suicidality has become an increasingly critical issue within public health, claiming approximately 700,000 lives globally each year and maintaining a suicide rate of 9.0 per 100,000 individuals worldwide (WHO, 2021). In the United States, it was the 12^th^ leading cause of death from 2010 to 2018 (Hedegaard *et al*., 2020). The economic impact of suicidal behaviour is profound, with an estimated cost of $70 billion annually in the U.S. alone (CDC, 2020). Research has highlighted the genetic basis of suicidality, complementing the roles of environmental and individual factors (Strawbridge *et al*., 2019; Li *et al*., 2023). For instance, monozygotic twins demonstrate a significantly higher likelihood of suicide attempts and completions compared to dizygotic twins (Li *et al*., 2023). Genome-wide association studies (GWAS) have identified a single nucleotide polymorphism (SNP) heritability of 3.5% in the UK Biobank and 6.8% in the International Suicide Genetics Consortium meta-analysis (Mullins *et al*., 2014; 2022).

Neuroimaging studies have indicated associations between changes in subcortical structures and suicidality risk (Campos *et al*., 2021; Kim *et al*., 2021; Yin *et al*., 2022). These findings align with the brain-centric diathesis-stress model of suicidal behaviour (Mann *et al*., 2020), suggesting brain changes contribute to suicide risk. However, conflicting reports exist, with some studies finding no significant association between suicidality and subcortical brain volume (Rentería *et al*., 2017). For instance, the ENIGMA-MDD consortium found no significant differences in subcortical regions among individuals with or without suicidal ideation or behaviour. Another study involving adolescents with major depressive disorder did not find a link between suicide attempts and subcortical alterations (Gifuni *et al*., 2021). Such discrepancies may stem from sample heterogeneity and the acute nature of suicidal behaviour.

Similarly, brain volume has been shown to possess a heritable component (Blokland *et al*., 2012). Twin studies have revealed genetic influences on both overall brain and subcortical volumes (Tramo *et al*., 1998; Pfefferbaum *et al*., 2000). Notably, GWAS have identified five genetic variants associated with the sizes of the putamen and caudate nucleus among seven subcortical brain regions (Hibar *et al*., 2015). More recent GWAS have discovered numerous genetic variants linked to brain morphometry (Satizabal *et al*., 2019). Despite these insights, the extent of shared genetic loci between suicidality and subcortical brain volume remains underexplored, and the common underlying features are not fully understood. Moreover, the genetic overlaps at the polygenic level are still inadequately comprehended. Genetic investigations may provide a clearer understanding of the overlapping psychopathology between suicidality and brain volume than imaging studies alone. In this study, we hypothesised that there may be a shared genetic aetiology underlying suicidality and altered subcortical brain volumes from a genome-wide perspective.

Recent research has proposed the existence of a genetic ‘p factor’, indicating shared genetic variance across various disorders, particularly psychiatric symptoms (Caspi *et al*., 2014); Sprooten *et al*., 2022). This conceptualisation suggests shared components in the underlying pathophysiology of mental disorders, potentially explaining their comorbidity. Utilizing large-scale GWAS datasets on suicidality and subcortical brain volume, this study aims to elucidate the shared genetic architecture between these phenotypes. We introduce a common factor model extending the genomic ‘p factor’ to include suicidality and subcortical brain volume through Genomic Structural Equation Modelling (Genomic Structural Equation Modeling (SEM)). We conducted variant-based and gene/pathway-specific GWAS meta-analyses to identify loci significantly associated with this common factor. Furthermore, we sought to uncover cross-disorder risk loci between subcortical brain volume and suicidality using our common factor-informed approach, aiming to elucidate shared molecular mechanisms.

## Materials and methods

### GWAS summary data

We acquired Genome-Wide Association Study (GWAS) summary statistics pertaining to Suicide or other intentional self-harm (SUIC) from the FinnGen Biobank (https://www.finngen.fi/en/access_results), as well as data on Thought Life Not Worth Living (TLNWL) and Ever Self-Harmed (ESH) from the United Kingdom Biobank/Neale lab, Attempted suicide (SA) from the study led by Erlangsen *et al*. (2020) which can be retrieved within the iPSYCH Biobank (Erlangsen *et al*., 2020). Additionally, summary-level data on seven subcortical brain volumes including Amygdala (AMY), Accumbens (ACC), Caudate (CAU), Hippocampus (HIP), Pallidum (PAL), Putamen (PUT), and Thalamus (THA) with the Intracranial Volume (ICV) were sourced from the ENIGMA2 study, accessible via the public database (http://enigma.ini.usc.edu/research/download-enigma-gwas-results/). All samples were of European ancestry, and comprehensive details regarding sample collection, genotyping, processing, quality control, and imputation procedures for each GWAS have been previously documented and briefly outlined (Hibar *et al*., 2015; Kurki *et al*., 2023). Details regarding the number of samples are outlined in Table 1.

**Table 1.** Summary information of the phenotypes of our study

Phenotype	#Case	#Controls	Sample size	Source
SUIC	1,361	341,138	342,499	FinnGen
TLNWL	NA	NA	117,291	United Kingdom Biobank/Neale lab
ESH	5,099	112,634	117,733	United Kingdom Biobank/Neale lab
SA	6,024	44,240	50,264	iPSYCH
Accumbens	NA	NA	13,112	ENIGMA2
Amygdala	NA	NA	13,160	ENIGMA2
Caudate	NA	NA	13,171	ENIGMA2
Hippocampus	NA	NA	13,163	ENIGMA2
Pallidum	NA	NA	13,142	ENIGMA2
Putamen	NA	NA	13,145	ENIGMA2
Thalamus	NA	NA	13,193	ENIGMA2
ICV	NA	NA	11,373	ENIGMA2

Upon retrieving data from the FinnGen database, we initiated a meticulous process of data refinement. Initially, we conducted data cleaning to ensure its quality and reliability. Duplicate Single Nucleotide Polymorphisms (SNPs) were removed, and we extracted SNPs with a Minor Allele Frequency (MAF) exceeding 0.01. Additionally, SNPs with conflicting alleles and those with missing information within the Genome-Wide Association Study (GWAS) summary statistics for each disorder were excluded from further analysis.

### SNP-based heritability and genome-wide genetic correlation

To gauge the portion of phenotypic variance attributable to common genetic variants, known as SNP-based heritability (h^2^ SNP), we employed univariate LD-score regression (LDSC) (Bulik-Sullivan *et al*., 2015). This method was implemented using the Genomic SEM R package (Grotzinger *et al*., 2019). We adhered to default LDSC settings for quality control processes, which involved filtering SNPs to HapMap3, excluding SNPs within the major histocompatibility complex (MHC) region, and removing SNPs with a MAF less than 1%. The defaults in LDSC were followed in the quality control (QC) processes for creating the genetic covariance (S) and sampling covariance (V) matrices. The MHC region, characterised by a complex gene network, often contains SNPs with disproportionately large effect sizes, thus necessitating its exclusion to prevent skewing results from heritability and genetic correlation studies, as well as in the genomic SEM analyses (Grotzinger *et al*., 2019). LD scores used in the analysis were derived from the 1000 Genomes European sample, limited to HapMap3 SNPs for reliable heritability estimates.

### Genomic structural equation modelling analysis

Genomic factor analysis was conducted using the Genomic SEM R package. Initially, a genomic exploratory factor analysis was performed to determine the optimal number of factors describing shared genetic variation. This informed subsequent genomic confirmatory factor analysis to estimate model parameters for fitting. We employed diagonally weighted least squares estimation due to its robustness when modelling traits with varying characteristics. Model fit was evaluated using established criteria for absolute fit, including the standardised root mean square residual (SRMR) with values ≤ 0.10 indicating moderate fit and SRMR ≤ 0.05 indicating good fit; comparative fit index (CFI) with values≥0.90 indicating moderate fit and CFI≥0.95 indicating good fit; and lower chi-square statistic with p-value less than 0.05 suggesting a precise match and greater fit (Grotzinger *et al*., 2019). We use this approach to derive a potential common factor model and thereafter perform GWAS meta-analyses at SNP, gene and sub-network levels encompassing the phenotypes detected within the potential common factor.

## Results

### SNP-based heritability and genome-wide genetic correlations

The heritability estimates presented in Table 2 were derived from our analysis and are expressed on the observed scale. For traits with lower-than-usual heritability estimates and higher standard errors compared to other studies, the SNP-based heritability could not be identified due to insufficient statistical power. Using bivariate LDSC (Bulik-Sullivan *et al*., 2015) implemented in the R package Genomic SEM, we estimated genetic correlations (rg) among the twelve traits. It is important to note that LDSC can sometimes provide estimates outside the range of -1 to + 1, particularly under conditions of large standard errors or highly significant genetic correlations between studies. Additionally, we were unable to generate a genetic correlation estimate for the amygdala with the other phenotypes due to its negative heritability estimate.

**Table 2.** Heritability estimates from our analysis

Trait	h^2^	h^2^(SE)	Z-score	Lambda GC	Mean Chi^2^	h^2^_inter	h^2^_inter_se	#SNPs for heritability analysis
SUIC	0.0025	0.0013	2.02	1.0292	1.0232	1.0065	0.07	1150849
TLNWL	0.0735	0.0054	13.7	1.1577	1.1788	1.0086	0.0072	1078769
ESH	0.0217	0.0044	4.96	1.0535	1.0613	1.0107	0.0066	1078769
SA	0.08	0.0122	6.6	1.0987	1.1107	1.0221	0.0091	922234
Accumbens	0.0905	0.0379	2.39	0.9967	1.0027	0.9796	0.0062	1171081
Amygdala	−0.0197	0.0322	−0.613	0.9944	0.9964	1.0014	0.0059	1171167
Caudate	0.2495	0.00397	6.28	1.0273	1.0326	0.9691	0.0062	1171185
Hippocampus	0.1542	0.0386	4	1.0103	1.0244	0.9844	0.0067	1171208
Pallidum	0.1551	0.0415	3.74	1.0065	1.0199	0.9799	0.0068	1171081
Putamen	0.2972	0.0481	6.17	1.0141	1.0268	0.951	0.0075	1171189
Thalamus	0.1314	0.0379	3.46	1.0065	1.0151	0.9816	0.0065	1171264
ICV	0.1845	0.00436	4.24	1.0383	1.0417	1.0008	0.0066	1171991

**Figure 1.**
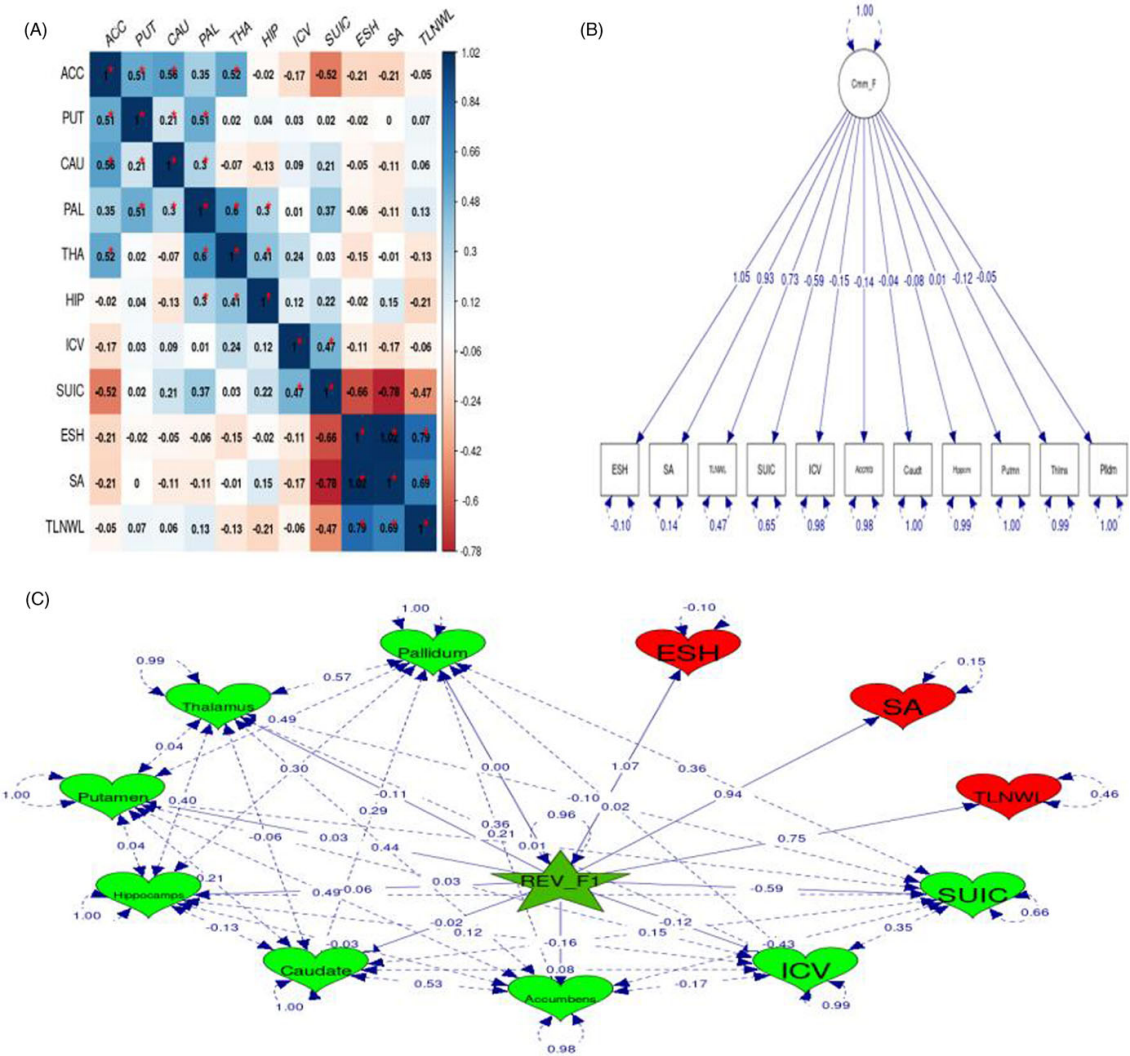
(**A**) The heatmap shows the genetic correlations (rg) between various brain structures and suicidality-related traits. The values represent the strength and direction of the genetic correlations, with significant (P-values less than 0.05) correlations indicated by asterisks (*). The colour scale ranges from blue (positive correlations) to red (negative correlations), with darker shades representing stronger correlations. (**B**) Path diagram for the single common factor model. This figure illustrates the overall common variance among all included traits. Ellipses represent latent variables, rectangles represent observed variables/traits, numbers on arrows are standardised factor loadings, and numbers at the ends of arrows are residual variances. (**C**) Path diagram of the revised common factor (Labelled ‘REV_F1’). This diagram illustrates the overall common variance among all included traits, representing observed variables with ‘heart’ shapes and the unobserved (latent) variable with a ’star’ shape. It suggests two groups of disorders sharing the same common factor: the first group in red and the second group in green. One-headed arrows represent regression connections between variables, while two-headed arrows indicate the variance of a variable or the covariance between a variable and itself. This analysis aimed to identify overlapping genetic factors and elucidate potential shared molecular mechanisms across the included traits.

Our study identified a marginal positive genetic correlation between SUIC and intracranial volume (ICV) (rg = 0.47; p-value = 0.024) and a negative genetic correlation (however not significant) between SUIC and accumbens (rg = -0.52; p-value = 0.93). (see Fig. 1A). There are notable positive genetic correlations between several subcortical brain volumes (e.g., accumbens, putamen, caudate, pallidum, thalamus). We found significant genetic correlations between the putamen (PUT) and accumbens (ACC) (rg = 0.51; p-value = 0.043), caudate (CAU) and accumbens (ACC) (rg = 0.56; p-value = 0.0168), thalamus (THA) and accumbens (ACC) (rg = 0.52; p-value = 0.022), and a strong positive genetic correlation between the pallidum (PAL) and thalamus (THA) (rg = 0.6; p-value = 0.02). Other Suicidality-Related Traits (ESH, SA, TLNWL) exhibit various correlations with each other and non-significant correlations with brain structures, with ESH and SA showing high significant correlations with each other (rg = 1.02; p-value = 2.45 × 10^-14^). The pattern of correlations highlights potential shared genetic underpinnings between certain brain volumes and suicidality, warranting further investigation into the underlying mechanisms. This analysis provides insight into the genetic architecture connecting brain structures and suicidality-related traits, suggesting both shared and unique genetic factors across these phenotypes.

### Genomic structural equation modeling analysis

First, we assessed the extent of common genetic variance among all included traits by evaluating the performance of a common genetic factor model. Although the model with freely determined loadings converged, it did not fit well (chisq(44) = 215.0768, Pchisq = 2.21 × 10^-24^, AIC = 259.0768, CFI = 0.696, SRMR = 0.194) (Fig. 1B).

Our genomic SEM analysis reveals an intriguing revised common factor model that fits the data well, with the best-fit statistics (chisq(15) = 25.69, Pchisq = 0.04, AIC = 127.69, CFI = 0.981, SRMR = 0.047) (Fig. 1C). This model identifies a common latent factor divided into two distinct groups of phenotypes. The first group includes the suicidal traits from the UK Biobank (ESH, SA, and TLNWL) highlighted in red, while the second group includes SUIC, ICV, Accumbens, Caudate, Hippocampus, Pallidum, Thalamus, and Putamen highlighted in green. This suggests that SUIC has a closer genomic link with subcortical brain volume and ICV compared to the suicidal traits ESH, SA, and TLNWL. However, both groups of traits exhibit the same genomic common factor, indicating the presence of a shared molecular mechanism.

### GWAS meta-analysis at variant and gene level

We conducted a variant-based GWAS meta-analysis using RE2C (v1.06) (Lee *et al*., 2017) to account for sample overlap among GWAS summary data. Significant variants were identified based on the RE2C P-value statistic (RE2C*P < 5 × 10^-8^). Variants that became significant after meta-analysis but did not reach genome-wide significance in individual trait GWAS datasets were considered novel (Kanai *et al*., 2016). In addition to the RE2C model, we performed cross-trait GWAS meta-analyses using both fixed effect (FE) and modified random effects (RE2) models (Han and Eskin, 2011), integrated into the METASOFT software (http://genetics.cs.ucla.edu/meta/). The FE model, which assumes the GWAS traits examined the same (fixed) effect, used the inverse variance weighted technique to estimate SNP meta-analysis statistics (effect size and p-value). In cases of heterogeneity, indicated by I^2^ statistics, METASOFT employed the RE2 model to estimate SNP meta-analysis statistics. Gene and subnetwork-specific meta-analyses were conducted using ancMETA (Chimusa and Defo, 2022), which incorporates summary GWAS information and aggregates SNPs within nearby genes. ancMETA provides information on significant genes and hub genes based on known biological protein-protein networks, shedding light on potential biological pathways shared across disorders. The meta-analysis GWAS focused on two sets of phenotypes:

**Group 1:** ESH, SA, and TLNWL.

**Group 2**: SUIC, ICV, Accumbens, Thalamus, Putamen, Caudate, Pallidum, and Hippocampus.

### GWAS meta-analysis at SNP level between ESH, SA, and TLNWL

Our cross-trait meta-analysis using the RE2C model identified 37 significant variants (RE2C*P < 5 × 10^-8^) (Table 3, Supplementary Table 1), all of which exhibited small effect sizes. Of these, 31 were novel, meaning they were not previously associated with any of the disorders (P_each_study_>5 × 10^-8^).

**Table 3.** Top significant variants from cross-trait meta-analysis between each set of phenotypes

ESH, SA and TLNWL												
variants	chr	bp	ref	alt	Nearest gene	P-ESH	P-SA	P-TLNWL	RE2C*P	Beta	SE	I^2^
rs17408393	18	53183611	A	G	*DCC*	2.85 × 10^-05^	0.49	9.41 × 10^-08^	1.53 × 10^-09^	0.0045	0.0008	88
rs62099230	18	53195342	G	A	*DCC*	3.49 × 10^-05^	0.49	1.09 × 10^-07^	2.14 × 10^-09^	0.0044	0.0008	88
rs17487277	18	53192574	C	G	*DCC*	3.7 × 10^-05^	0.49	1.1 × 10^-07^	2.25 × 10^-09^	0.0044	0.0008	88
rs62100771	18	53214760	A	G	*DCC*	7.99 × 10^-05^	0.48	5.89 × 10^-08^	2.79 × 10^--09^	0.0043	0.0008	88
rs150002680	15	83579597	A	G	*SH3GL3*	7.9 × 10^-09^	0.77	0.166	2.86 × 10^-09^	0.024	0.004	0

SUIC, ICV, accumbens, caudate, hippocampus, pallidum, thalamus, and putamen									
variants	chr	bp	ref	alt	Nearest gene	RE2C*P	Beta	SE	I^2^
rs6567261	18	62184009	T	C	*PIGN*	5.68 × 10^-30^	−0.0012	0.0004	92.77
rs11754988	6	74253222	C	T	*CD109*	5.17 × 10^-27^	−0.001	0.000385	92.3
rs56161836	1	245197588	G	A	*KIF26B*	6.69 × 10^-27^	−0.001	0.000378	92.31
rs13022308	2	166492125	G	A	*SCN7A*	7.94 × 10^-27^	−0.001	0.000376	92.2
rs2302862	3	10216809	T	C	*IRAK2*	8.9 × 10^-27^	−0.001	0.000379	92.2

Our SNP-level results indicate that the most significant variants are located within the *DCC* gene. Additionally, associations were found with SNPs in the *SH3GL3*, *STIM2*, *MEAF6*, and *RSPO1* genes (Table 3, Supplementary Table 1). The top four significant novel loci are all located within the *DCC* gene, while other new associations were found within the *STIM2*, *MEAF6*, and *RSPO1* genes. These findings highlight new genetic loci that add value to previously identified genes in the literature.

### GWAS meta-analysis at SNP level between SUIC, ICV, accumbens, caudate, hippocampus, pallidum, thalamus, and putamen

**Figure 2.**
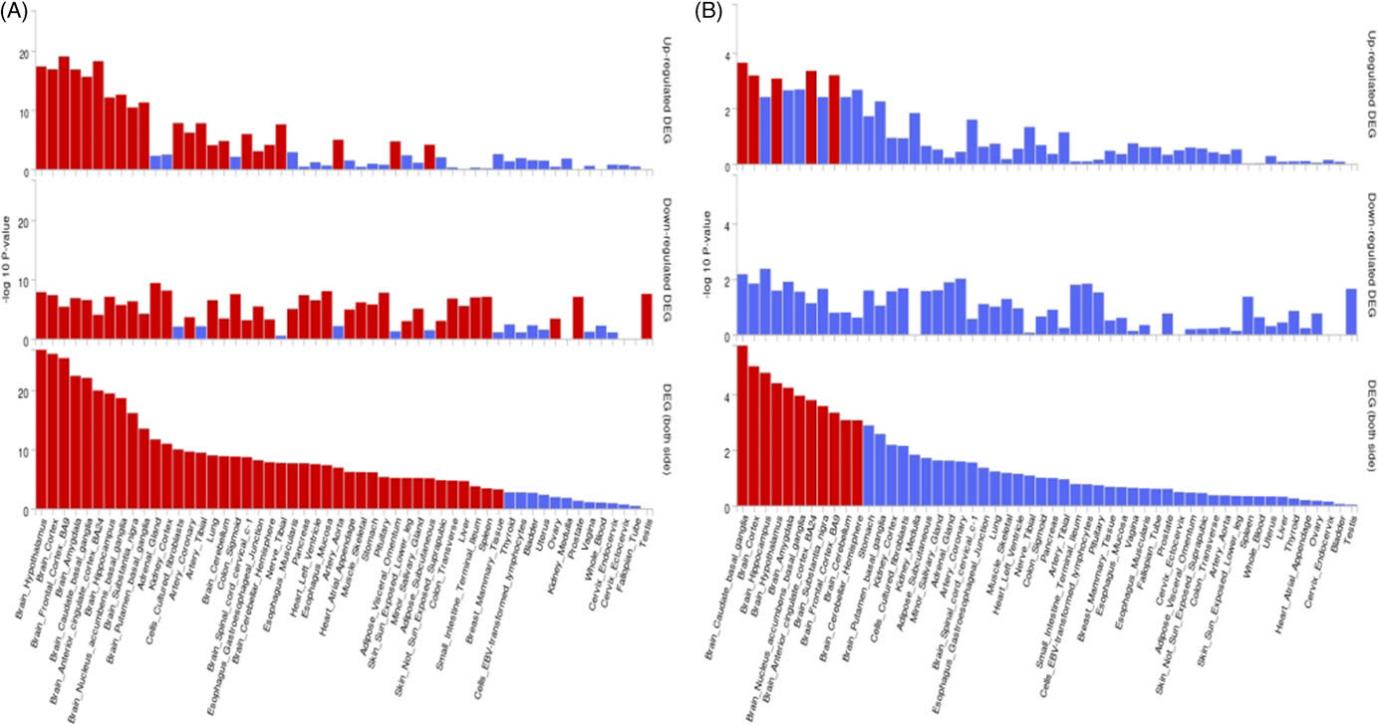
(**A)**- Bar plot showing enrichment tissues of all the nearby genes from significant cross-associated SNPs; (**B**) bar plot showing enrichment tissues of nearby genes from the significant potential pleiotropic (accumbens, caudate, hippocampus, pallidum, thalamus, and putamen combined) SNPs. The red colour speaks for significance and the blue one speaks for non-significance.

Our GWAS meta-analysis using the RE2C model identified 484 significant variants, all exhibiting low effect sizes (Table 3, Supplementary Table 2). Among these, 64 SNPs showed potential pleiotropic effects, influencing multiple subcortical brain structures simultaneously, including the accumbens, caudate, hippocampus, pallidum, thalamus, and putamen. According to the FUMA analysis, the genes located near these significant loci exhibit enrichment across various brain regions. The highest levels of enrichment were found in the hypothalamus, brain cortex, and frontal cortex (Fig. 2A). However, the genes near the pleiotropic loci showed enrichment in nearly all parts of the brain, with the exception of the putamen basal ganglia and the spinal cord (Fig. 2B).

These findings suggest widespread genetic influences across various brain regions, emphasising the importance of considering multiple brain structures when studying genetic associations with SUIC and subcortical brain volumes.

### GWAS meta-analysis at gene and sub-network level between ESH, SA, and TLNWL

#### Gene-level analysis

At the gene level, ancMETA identified 893 significant genes (Table 3; Supplementary Table 3) associated with ESH, SA, and TLNWL (overall *P* < 0.05). The top significant genes include *RANBP17* (p-value = 3.25 × 10^-05^), *C6orf89* (p-value = 1.8 × 10^-04^), and *GPHN* (p-value = 2.1 × 10^-04^). *RANBP17* is Located on chromosome 5q35.1 and encodes RAN-binding protein 17, a nuclear transport receptor. It has been associated with the severity of suicide attempts in mood disorders at the polymorphism level (Zai *et al*., 2021). *C6orf89* which encodes the bombesin receptor-activated protein (BRAP), is associated with allergic rhinitis and asthma and is potentially implicated in the stress response of lung epithelia (Liu *et al*., 2016; Xu *et al*., 2017). Studies in mice suggest that BRAP regulates dendritic spine development and synaptic plasticity in the hippocampus, providing a protective behavioural response to stress (Yao *et al*., 2023). Regarding *Gephyrin* (*GPHN),* previous studies have linked exonic microdeletions in this gene to neurodevelopmental issues such as idiopathic generalised epilepsy (Dejanovic *et al*., 2014), schizophrenia, autism spectrum disorder, and epileptic seizures. These findings suggest that while the effects of variants within these genes differ between studies, the aggregation of variant effects within these genes significantly contributes to the cross-phenotype association of ESH, SA, and TLNWL.

#### Sub-network level analysis

**Figure 3.**
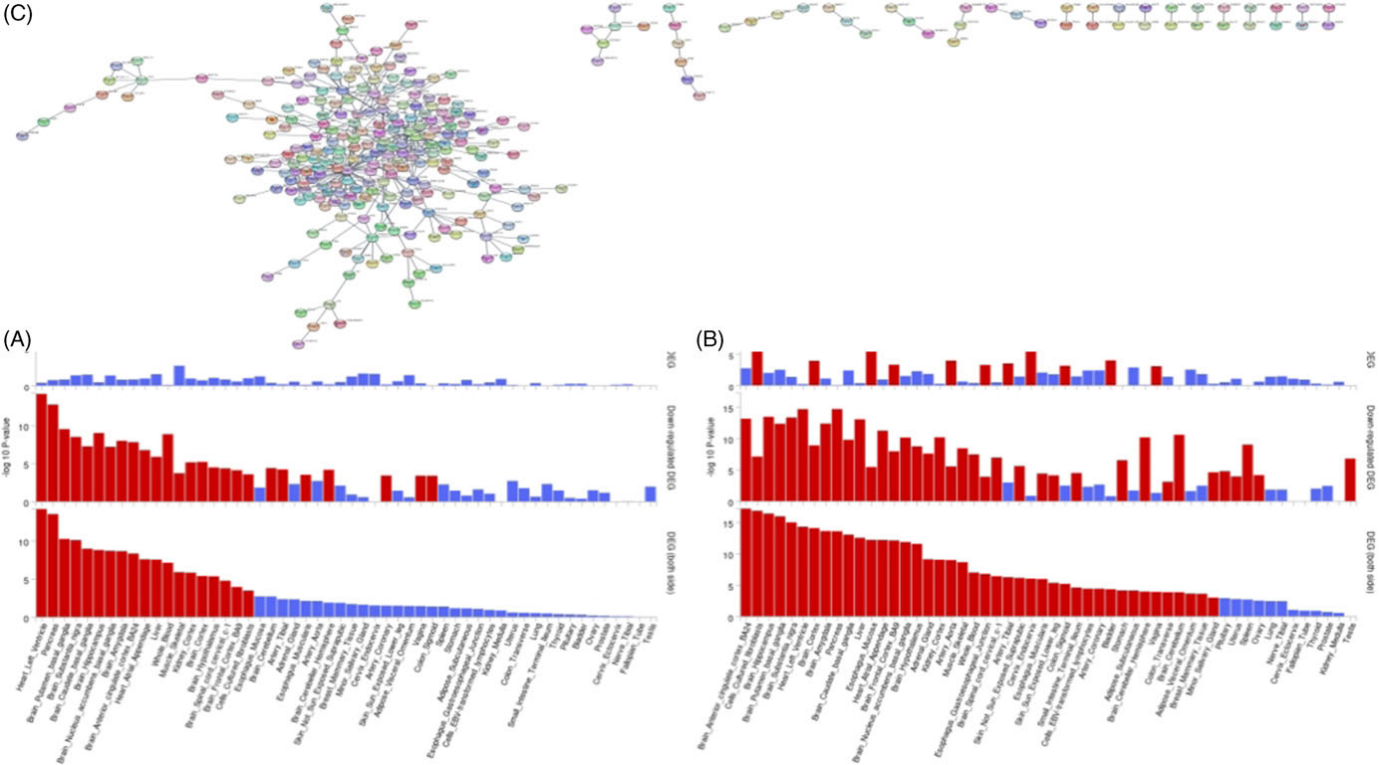
**(A)** The bar plot shows tissue enrichment for all significant genes and hub genes identified through ancMETA analysis at the gene and subnetwork levels, using suicidality data from FinnGen and subcortical brain volume data from ENIGMA. (**B)** The bar plot displays tissue enrichment for significant genes and hub genes identified through ancMETA analysis at the gene and subnetwork levels, using emotional stability, social anxiety, and tolerance to noise and workload data from the UK Biobank. Red indicates significance, while blue indicates non-significance. (**C)** This potential subnetwork includes all significant genes and hub genes combined, generated by ancMETA from the two sets of phenotypes.

At the sub-network level, ancMETA identified 50 significant hub genes (Supplementary Table 4). Among these, the top significant genes were *GPHN* (p-value = 0.00022), *RGS2* (p-value = 0.004), and *ATP1A1* (p-value = 0.0045). These hub genes indicate that the aggregation’s effect of variants within these genes significantly contributes to the cross-phenotype association at the pathway/gene set level, encompassing the phenotypes of ESH, SA, and TLNWL. Our FUMA analysis revealed that the significant genes and hub genes identified in our ancMETA results showed significant expression enrichment across all brain regions. The top enriched tissues include the brain anterior cingulate cortex, cultured fibroblast cells, brain hippocampus, brain putamen basal ganglia, and brain substantia nigra (Fig. 3B).

Previous studies have highlighted the association of numerous variants within the *RGS2* gene with a higher risk of successful suicide (Cui *et al*., 2008; Amstadter *et al*., 2009). *ATP1A1*, a member of the sodium/potassium pump (Na+/K+-ATPase) family expressed in the brain, regulates the gradient of potassium and sodium across cellular membranes (Richards *et al*., 2007). Research has verified the involvement of brain Na+/K+-ATPase α subunit isoforms, particularly the α2 and α3 subunits, in various behavioural features, linking them to mental and behavioural disorders in humans (Lingrel *et al*., 2007; Tochigi *et al*., 2008). Another study has demonstrated the connection between ATP1A1 expression levels and clinical anxiety scores in patients with major depressive disorder (Zhao *et al*., 2016).

### GWAS meta-analysis at gene and sub-network level between suic, icv, accumbens, caudate, hippocampus, pallidum, thalamus, and putamen

#### Gene-level analysis

In a comprehensive GWAS meta-analysis, ancMETA identified 402 significant genes cross-associated with SUIC, ICV, and various subcortical brain regions, including the accumbens, caudate, hippocampus, pallidum, thalamus, and putamen (overall *P* < 0.05; Table 4, Supplementary Table 5). The top significant genes were *RPL11* (p-value = 1.8 × 10^-4^), *DDX4* (p-value = 4.03 × 10^-4^), and *WDR55* (p-value = 1.06 × 10^-3^). *RPL11* has been previously implicated in the ribosomal pathway, playing a role in the pathogenesis of mild cognitive impairment and Alzheimer’s disease (Qin *et al*., 2023). It is also associated with brain arteriovenous malformations (Zhang *et al*., 2021) and has been proposed as a biomarker for major depressive disorder Zhang *et al*., 2020) and low-risk neuroblastoma (Nguyen *et al*., 2011). *WDR55* encodes WD repeat-containing protein 55, which modulates ribosomal RNA biogenesis, cell cycle progression, and organ development. It has been identified as a significant CpG site and methylated region associated with depression risk in Chinese monozygotic twins (Wang *et al*., 2021).

**Table 4.** Top 3 significant genes and subnetwork hub genes from cross-trait meta-analysis between each set of phenotypes

SUIC, ICV, accumbens, caudate, hippocampus, pallidum, thalamus, and putamen												
Gene	#Study	Overall P	Q	P_Q	P_SUIC	P_Cau	P_ICV	P_Acc	P_Tha	P_Pal	P_Put	P_Hip
*RPL11*	8	1.8E-04	6.68	0.46	0.012	0.001	0.002	0.00046	0.0006	0.00028	0.001	0.00041
*DDX4*	8	4.0E-04	5.34	0.62	0.01	0.00032	0.014	0.00087	0.00064	0.00033	0.000386	0.00052
*WDR55*	8	1.06E-03	6.61	0.47	0.0036	0.00046	0.012	0.00062	0.0093	0.00035	0.0012	0.001

Sub-network Hub	#Study	Overall P	Q	P_Q	P_SUIC	P_Cau	P_ICV	P_Acc	P_Tha	P_Pal	P_Put	P_Hip
*NEB*	8	0.012	7.28	0.4	0.44	0.08	0.164	0.198	0.06	0.052	0.0086	0.056
*EEF1D*	8	0.0138	6.125	0.52	0.24	0.066	0.433	0.033	0.139	0.0372	0.42	0.115
*B2M*	8	0.019	6.53	0.4791	0.35	0.0869	0.236	0.415	0.081	0.062	0.0769	0.0394

ESH, SA and TLNWL								
Gene	#Study	Overall P	Q	P_Q	I^2^	P_ESH	P_SA	P_TLNWL
*RANBP17*	3	3.25E-05	1.55	0.46	0	0.0021	0.225	0.000236
*C6orf89*	3	1.84E-04	1.55	0.46	0	0.00029	0.031	0.000229
*GPHN*	3	2.1E-04	1.08	0.58	0	0.00022	0.0094	0.00262

Sub-network Hub	#Study	Overall P	Q	P_Q	I^2^	P_ESH	P_SA	P_TLNWL
*GPHN*	3	2.1E-04	1.08	0.58	0	0.022	0.396	0.22
*RGS2*	3	0.00395	1.55	0.46	0	0.037	0.391	0.378
*ATP1A1*	3	0.00452	1.557	0.459	0	0.0488	0.203	0.04573

#### Sub-network level analysis

At the sub-network level, ancMETA identified 22 significant hub genes (Supplementary Table 6). The most significant hub genes included *NEB* (p-value = 0.012), *EEF1D* (p-value = 0.013), and *B2M* (p-value = 0.019) (Table 4). These hub genes suggest that the aggregate effect of variants within these genes significantly contributes to the cross-phenotype association risk at the pathway/gene set level, encompassing SUIC and brain structures such as ICV, accumbens, caudate, hippocampus, pallidum, thalamus, and putamen. Our FUMA analysis showed that the significant genes and hub genes identified from ancMETA results exhibited significant expression enrichment in all brain regions, except for the cerebellum and cerebellar hemisphere, where down-regulated expressed genes were specific. The top enriched tissues included the heart and left ventricle, pancreas, putamen basal ganglia, substantia nigra, and hippocampus (Fig. 3A).

*NEB* (on chromosome 2q23.3) encodes nebulin, a protein extensively expressed in skeletal muscle, known for regulating muscle contraction and stabilising thin filaments (Chandra *et al*., 2009). Immunohistochemistry has shown nebulin expression predominantly in the cytoplasm of pyramidal neurons and subcortical endothelial cells in the adult brain (Laitila *et al*., 2012). Whole exome sequencing identified two likely pathogenic *NEB* variants in a patient with cognitive impairment and dysmorphic features (Nóbrega *et al*., 2024), suggesting a potential role for nebulin in the central nervous system and suicidality risk.

*EEF1D* (located on chromosome 8q24.3) undergoes alternative splicing in the brain and testis, affecting its expression. Mutations in EEF1D have been linked to neurodevelopmental disorders, microcephaly, and severe intellectual disability (Kaitsuka and Matsushita, 2015; McLachlan *et al*., 2019). *B2M* (Beta-2-Microglobulin) has been identified as a biomarker for stress-related disorders, including suicide (Le-Niculescu *et al*., 2020). Additionally, *B2M* is associated with various neuropsychiatric phenotypes, such as alcoholism, autism, depression, eating disorders, pain, and ageing, potentially mediating the effects of stress in these conditions (Le-Niculescu *et al*., 2020).

### Utilizing network and pathway analysis across two sets of disorders

In this study, we performed a network and pathway analysis involving two distinct sets of disorders. Initially, we used ancMETA to generate subnetworks containing significant genes and hub genes for each set of disorders. These subnetworks were then merged using Cytoscape version 3.7.2 (see Fig. 3C). We conducted pathway enrichment analysis based on Gene Ontology (GO), Reactome pathways, and the Protein-Protein Interaction network and visualised the results with the StringApp plugin in Cytoscape version 3.7.2 (Shannon *et al*., 2003; Doncheva *et al*., 2019). The merged subnetwork of genes was assessed for enrichment in pathways and gene ontology using the Cytoscape plugin StringApp.

In the resulting network, we identified a significant number of pathways (FDR<0.05), specifically 132 Reactome pathways, 50 KEGG pathways, and 51 WikiPathways. The most notable KEGG pathways included the Rap1 signalling pathway (FDR = 1.3 × 10^-4^), osteoclast differentiation (FDR = 1.3 × 10^-4^), T cell receptor signalling pathway (FDR = 1.3 × 10^-4^), and viral carcinogenesis (FDR = 3.9 × 10^-4^). Reactome analysis highlighted significant pathways such as Disease (FDR = 1.12 × 10^-10^), signalling by receptor tyrosine kinases (FDR = 3.9 × 10^-9^), signal transduction (FDR = 2.95 × 10^-7^), adaptive immune system (FDR = 1.5 × 10^-6^), infectious disease (FDR = 4.01 × 10^-6^), and the immune system (FDR = 1.1 × 10^-5^). WikiPathways analysis identified VEGFA-VEGFR2 signalling (FDR = 6.4 × 10^-8^), RANKL/RANK signalling pathway (FDR = 7.97 × 10^-7^), and the T-cell receptor signalling pathway (FDR = 5.07 × 10^-5^) as particularly significant.

A detailed table listing each significant pathway per database, along with all significant GO biological processes, components, and functions, is provided in Supplementary Tables 7-13. Additionally, we compiled a list of pathogenic loci identified from our gene/subnetwork GWAS meta-analysis using ancMETA on the two sets of phenotypes, with pathogenic criteria based on a probability of being ‘loss-of-function Intolerant’ > 0.9 (Lek *et al*., 2016) (Supplementary Table 14).

## Discussion

The findings of this study offer crucial insights into the intricate genetic relationship between suicidality and alterations in brain structure, particularly in subcortical brain regions. This highlights possible shared molecular mechanisms and genetic underpinnings. The discovery of a common genetic factor between suicidality and subcortical brain regions underscores the existence of shared pathways and biological processes. Although we identified a nominal positive genetic correlation between SUIC and ICV, this emphasises the complexity of the relationship and the need for further exploration using diverse methodologies and larger sample sizes (Franke *et al*., 2016; Smeland *et al*., 2018). Furthermore, our study demonstrated a common factor emerging from two cohorts: the suicide cohort from the UK Biobank (emotional stability, social anxiety, and tolerance to noise and workload) and the phenotypes, including SUIC from FinnGen and subcortical brain volume data. This suggests a direct overlap between SUIC and subcortical brain regions in the FinnGen cohort, compared to the UK Biobank cohort.

At the SNP level, our comprehensive analysis revealed significant variants within key genes, including *DCC*, *SH3GL3* (rs150002680), *STIM2* (rs28592695), *MEAF6* (rs6682470), and *RSPO1* (rs115632986) from the UK Biobank. This adds to the loci previously reported by Strawbridge and colleagues (2019). The SNP-based GWAS meta-analysis between SUIC and subcortical brain volume identified 484 significant variants with low effects, with 64 SNPs showing potential pleiotropic effects on the accumbens, caudate nucleus, hippocampus, pallidum, thalamus, and putamen. These findings highlight the interconnectedness of genetic factors and support previous research linking suicidality to frontal-subcortical circuits (Tekin and Cummings, 2002; Dobbertin *et al*., 2023).

Beyond individual variants, our gene and subnetwork GWAS meta-analysis unveiled numerous significant genes and hub genes implicated in both SUIC and altered brain volume. Particularly noteworthy are the loss-of-function-related genes, which indicate a pathogenic potential and heightened risk for suicidality (refer to Supplementary Table 14).

The integration of these genetic findings into a comprehensive network analysis revealed enriched functionalities across various biological processes and pathways. Notably, genes related to neuroinflammation were significantly enriched, with pathways involving immune signalling, apoptosis, nervous system, neurodevelopmental disorders (such as Alzheimer’s and Huntington’s Disease), infectious diseases, and neurotrophic factors. These findings suggest potential targets for therapeutic intervention. Several pathways and GO enrichment strategies identified in our study align with previous findings that link the blood-brain barrier and suicidal risk (Mann and Risk, 2020; Pandey and Dwivedi, 2012; Wisłowska-Stanek *et al*., 2021; Bengoechea-Fortes *et al*., 2023).

Our findings indicate that the presence and severity of suicidality are associated with an inflammatory signature detectable in both blood and brain tissues. This suggests a biological continuity underlying suicidality, potentially indicating a common heritability. These results support the role of brain and peripheral blood inflammation in suicide risk. Our findings suggest that these hub genes or enriched common pathways underlying shared molecular mechanisms between suicidality and altered subcortical brain volume could mean that treatments targeting these biological enriched pathways would have broad-spectrum therapeutic effects, improving precision medicine and personalized therapeutic development in suicidal individuals.

The identification of genes involved in the dysregulation of the blood-brain barrier and immune function underscores the bidirectional communication between the brain and peripheral immune system in the context of suicidal risk. These results are corroborated by previous studies, further strengthening the validity of our findings and highlighting potential translational implications (Sun *et al*., 2024). Our findings expand on previous research that identified genes substantially expressed in brain tissue and enriched in pathways related to immunologic markers, cellular stress response, gene regulation, and DNA repair (Docherty *et al*., 2023; Diblasi *et al*., 2021; Sokolowski and Wasserman, 2020).

The involvement of glial cells and microglia in inflammatory responses within the central nervous system (Yang and Zhou, 2019) provides mechanistic insights into the pathophysiology of suicidality and altered brain volume. Glial cells, the most prevalent cells in the central nervous system, interact with immune system cells, neurons, and brain microvascular endothelial cells. Microglia, in particular, are resident innate immune cells. Studies have shown higher densities of activated microglia (Schnieder *et al*., 2014) in the white matter of suicide postmortem cases, as well as higher microglial priming and macrophage recruitment (Torres-Platas *et al*., 2014). The transmission of inflammatory signals from the periphery to the brain via humoral transmigration or sensory afferent projections through the blood-brain barrier can stimulate microglial activation (Dantzer, 2009; Serna-Rodríguez *et al*., 2022), and suicidality has been linked to anomalies in endothelial cells and the blood-brain barrier (Pantazatos *et al*., 2017; Greene *et al*., 2020). The identified hub genes and potential significnt pathways related to anti-neuroinflammation and immune regulation offers a promising approach to treating suicidal behaviour with altered subcorticalbrain volume, which is frequently impacted by complicated neuroimmune interactions. However, converting these techniques into clinically effective medicines necessitates overcoming obstacles in gene delivery, safety, and selectivity. Ongoing research in neuroinflammation, immunological signalling, and gene therapy technologies could show promise for more customised and effective therapies for people at risk of suicidality.

Despite these significant findings, several limitations warrant consideration. Generalising our findings to other populations and ethnicities requires replication in diverse cohorts. This study only included individuals of European ancestry, limiting the generalizability of the findings. Expanding future analyses to include diverse populations is essential for broader applicability. Additionally, the reliance on GWAS summary statistics and the inherent statistical power of the original studies necessitates cautious interpretation of null results. Therefore, null conclusions in our research do not always imply a lack of association. The negative heritability of the amygdala did not provide a clear picture of genetic correlation and was not included in our SNPs, gene, and subnetwork GWAS meta-analysis. A well-powered GWAS of altered brain volume and suicidality could improve the detection of significant variants, genes, and pathways shared between these traits. Most of our suicide phenotypes have very low heritability, confirmed by the low effect sizes of relevant loci. Hence, the identified loci, networks, and functional pathways need validation in future studies with additional experiments, either in vivo or in vitro. Our findings are limited to autosomal chromosomal common variants. Copy number variants and other rare variants independently demonstrate strong penetrance for suicidal risk (Gross *et al*., 2015). Incorporating these in future studies could provide a more comprehensive view of genetic contributions. Known sex-specific effects in individuals with suicidal behaviour (Kia-Keating *et al*., 2007; Powers *et al*., 2020) and brain development (Mallard *et al*., 2021) further necessitate future studies on rare variants and sex-specific shared mechanisms.

In conclusion, this study represents a pioneering effort in elucidating the shared genetic architecture of suicidality and subcortical brain volumes. By uncovering overlapping genetic factors and biological pathways, we provide novel insights into the complex interplay between brain structure and suicidal behaviour. These findings hold promise for developing targeted interventions and personalized treatment strategies aimed at mitigating suicidality in vulnerable individuals. Further research exploring rare variants, sex-specific effects, and functional validations will be crucial for advancing our understanding of these complex phenomena and informing clinical practice.

## Supporting information

Defo and Ramesar supplementary material Defo and Ramesar supplementary material

## Supplementary material

The supplementary material for this article can be found at https://doi.org/10.1017/neu.2025.12.
